# The Relationship Between Perceived Corporate Social Responsibility and Employee-Related Outcomes: A Meta-Analysis

**DOI:** 10.3389/fpsyg.2021.607108

**Published:** 2021-07-08

**Authors:** Agnieszka Paruzel, Hannah J. P. Klug, Günter W. Maier

**Affiliations:** Work and Organizational Psychology, Department of Psychology, Bielefeld University, Bielefeld, Germany

**Keywords:** commitment, job satisfaction, meta-analysis, organizational citizenship behavior, corporate social responsibility, identification

## Abstract

Although there is much research on the relationships of corporate social responsibility and employee-related outcomes, a systematic and quantitative integration of research findings is needed to substantiate and broaden our knowledge. A meta-analysis allows the comparison of the relations of different types of CSR on several different outcomes, for example to learn what type of CSR is most important to employees. From a theoretical perspective, social identity theory is the most prominent theoretical approach in CSR research, so we aim to investigate identification as a mediator of the relationship between CSR and employee-related outcomes in a meta-analytical mediation model. This meta-analysis synthesizes research findings on the relationship between employees' perception of CSR (people, planet, and profit) and employee-related outcomes (identification, engagement, organizational attractiveness, turnover (intentions), OCB, commitment, and job satisfaction), thereby distinguishing attitudes and behavior. A total of 143 studies (*N* = 89,396) were included in the meta-analysis which was conducted according to the methods by Schmidt and Hunter (except of the meta-analytical structural equation model). Mean effect sizes for the relationship between CSR and employee-related attitudes and behaviors were medium-sized to large. For attitudes, the relationships were stronger than for behavior. For specific types of CSR, average effect sizes were large. Identification mediated the relation between CSR and commitment, job satisfaction, and OCB, respectively. Based on our results, we give recommendations concerning the design of CSR initiatives in a way that benefits employees.

## Introduction

Corporate social responsibility (CSR) initiatives are already well-established in companies worldwide (KPMG International, 2015). CSR is not only contributing to the welfare of our society, but is also associated with financial performance (Orlitzky et al., 2003), a good company reputation (Aguinis and Glavas, 2012), influences consumers' buying decisions (Fatma and Rahman, 2015) as well as customer commitment (Ahmed et al., 2020) and is evaluated positively by employees (Rupp and Mallory, 2015). Micro-CSR, which is the psychological study of how CSR affects individuals, often-times specifically employees (Rupp and Mallory, 2015), gains more and more attention and is strongly demanded in research (Aguinis and Glavas, 2012; Rupp and Mallory, 2015; Gond et al., 2017).

In micro-CSR research, it is already well-known from literature reviews that CSR is positively associated with engagement, job satisfaction, commitment, and organizational citizenship behaviors (OCB), and negatively related to turnover intentions and actual turnover (e.g., Rupp and Mallory, 2015; Glavas, 2016; De Roeck and Maon, 2018).

However, some questions remain unanswered. First, it is unknown what types of CSR initiatives are most important to employees—initiatives benefitting themselves, environmental programs, or initiatives with a benefit for the society as a whole. Some researchers argue that for employees, initiatives directed toward themselves may be most important, others argue that employees tend to value initiatives with a benefit for the society as a whole (Farooq et al., 2017). A meta-analysis allows a quantification of the relationships of different types of CSR and employee-related outcomes. Second, it remains unknown which employee-related outcomes are most important to employees. This is relevant for practice, as companies may adjust their communication strategy of CSR to their employees to increase, e.g., commitment. We investigate the relationship of CSR and identification, engagement, commitment, job satisfaction, attractiveness to potential employees, turnover (turnover intentions and actual turnover), and organizational citizenship behavior as employee-related outcomes. These constructs are derived from Aguinis and Glavas' (2012) comprehensive review on an analysis of CSR on the organizational, institutional, and individual level. Third, reported correlations vary in size, for example for commitment from *r* = −0.01 and *r* = 0.11 (Vitell et al., 2010; Pérez et al., 2018) to *r* = 0.75 and *r* = 0.77 (Lee et al., 2013; Vlachos et al., 2014). Using a meta-analysis, our knowledge about the magnitude of relationship will gain in certainty. Finally, the role of identification remains unclear. Social identity theory (Ashforth and Mael, 1989), which is the most common theoretical background in CSR research on the individual level (Gond et al., 2017), suggests identification to explain how the positive effect of CSR on employees unfolds, therefore, it should be investigated as a mediator. In our meta-analysis, we determine the magnitude of the relationship of CSR and identification, and then test identification as a mediator of CSR and other employee-related outcomes such as commitment and job satisfaction. The main goal of this meta-analysis is to examine the relationship of different foci of CSR and employee-related outcomes. Another goal is to test identification as a mediator meta-analytically according to social identity theory (Ashforth and Mael, 1989).

### Corporate Social Responsibility

According to the triple bottom line of sustainability by Elkington (1994) three domains have to be taken into account simultaneously by an organization to be sustainable: people, planet and profit. The triple bottom line has transferred to CSR and is now widely used within the field (Aguinis, 2011; Swanson and Orlitzky, 2017). We hypothesize that different types of CSR—specifically CSR that focuses on people, the planet or profit—have different impacts on employee-related attitudes and behaviors. The term *people*, corresponds to a social focus of CSR, meaning that CSR is aiming at improving the welfare of society (Bergmans, 2006). As we focus on employees, we distinguish between *people-employee* (focus on an organization's own employees) and *people-society* (focus on society in general). *Planet* refers to a focus on the natural environment, aiming for ecological quality (Bergmans, 2006). *Profit* reflects an economic focus, adding value to economic prosperity (Bergmans, 2006). The profit category includes acting financially profitable, lowering costs and paying taxes, but can also include corporate donations. There are also alternative models and CSR concepts (Wartick and Cochran, 1985; Carroll, 1991; Wood, 1991; Turker, 2009; El Akremi et al., 2018), which can be integrated into the triple bottom line.

### Theoretical Background

Researchers rely on a variety of psychological theories to explain the association of CSR and employee-related outcomes. According to a review by Gond et al. (2017), social identity theory was the most widely used theory to explain working mechanisms of CSR on employee-related attitudes and citizenship behaviors. Other theoretical frameworks build upon fairness (Rupp et al., 2006) by regarding CSR as third-party justice observations. Employees perceiving CSR witness third parties—the beneficiaries of CSR—being treated fairly and assume that the company would also treat them fairly. Following similar assumptions, signaling theory explains how job applicants perceive CSR as a signal how their future working conditions in a company will be (Rynes, 1991). Others argue that working for a socially responsible company makes work more meaningful by contributing to the welfare of society (Aguinis and Glavas, 2019). For an overview of theoretical frameworks, see Rupp and Mallory's review (2015).

To formulate a research question and derive hypotheses, we rely on social identity theory because it is not only relevant for one specific outcome but can also explain the relationships of CSR and the other employee-related attitudes and citizenship behavior under investigation. According to De Roeck and Delobbe (2012), identification is a fundamental psychological process explaining why CSR can change organizational attitudes. Social identity theory (Tajfel and Turner, 1979) proposes that people make self-definitions based on social category memberships. For example, a basic social category is gender or profession. Later, this theory has been applied to the organizational context and this specific form of social identification is organizational identification (Ashforth and Mael, 1989). Moreover, people strive to identify with favorable social categories which are able to enhance their self-esteem (Tajfel, 1978; Hogg and Turner, 1985). A company's engagement in CSR is supposed to be a favorable and prestigious social attribute (Peterson, 2004; Brammer et al., 2007; Turker, 2009).

Three factors determine the extent to which employees develop a feeling of belongingness to their organization (Ashforth and Mael, 1989): distinctiveness, prestige, and salience of the out-groups. Distinctiveness is the uniqueness of values and practices of a group compared to other groups (Oakes and Turner, 1986), prestige designates the company's perceived prestige, and salience of out-groups increases the awareness of one's in-group (Ashforth and Mael, 1989). This means that employees' identification with their company increases if their CSR initiatives and programs are distinct and prestigious. When employees become aware of other companies' engagement in CSR, this simultaneously increases the awareness of CSR in their own company. De Roeck et al. found that the mere presence of CSR, the fact that a company engages in CSR, which means that employees not necessarily have to participate in CSR, increases identification, mediated by prestige (De Roeck et al., 2016).

*Hypothesis 1*: CSR is positively related to identification.

Apart from identification, social identity theory can also explain the relationship between CSR and other attitudes such as engagement which is characterized by a positive, fulfilling, work-related state of mind that is characterized by vigor, dedication and absorption (Schaufeli et al., 2006). Engaged employees are energized and enthusiastic about their work. Dedication is especially characterized by a strong involvement in work and the experience of a sense of significance (Bakker and Demerouti, 2008), elicited by distinct CSR initiatives and programs. Distinctiveness and prestige of CSR initiatives lead to a sense of significance, and, ultimately, engaged employees. The relationship of CSR and engagement was investigated several times (e.g., Gupta, 2017; Gao et al., 2018).

*Hypothesis 2*: CSR is positively related to engagement.

Concerning attractiveness to potential employees, which is an applicant's willingness to pursue jobs and to accept job offers in an organization (Tsai and Yang, 2010), we hypothesize that prospective employees strive for a membership in a socially responsible company. This membership is supposed to enhance their self-esteem (Smith and Langford, 2011). The prestige due to the company's engagement in CSR leads to the company being perceived as attractive to potential employees. The relationship of CSR and attractiveness was investigated several times (e.g., Kroh, 2014; Hong and Kim, 2017).

*Hypothesis 3*: CSR is positively related to attractiveness.

Actual turnover or turnover intentions are negatively related to CSR; we hypothesize that incumbent employees appreciate their companies' CSR and are not willing to leave their socially responsible employer. The negative relationship of CSR and turnover intentions (e.g., Low et al., 2017; Wang et al., 2017) or actual turnover (e.g., Bode et al., 2015; Ng et al., 2019) has been reported several times, and identification mediated this relationship (Lee et al., 2008; Wang et al., 2017; Islam et al., 2018).

*Hypothesis 4*: CSR is negatively related to turnover (actual turnover and turnover intentions).

Organizational commitment consists of three components (Allen and Meyer, 1990; Meyer and Allen, 1997): Employees are committed to their organization due to an emotional bond (affective commitment), due to moral-ethical reasons (normative commitment) or due to cost avoidance resulting from job change (continuance commitment) (Meyer and Allen, 1991). As social identity theory suggests, CSR is associated with organizational identification. Therefore, self-esteem derived from this membership will lead to an emotional bond (affective commitment). Moreover, as the company makes social investments, the employees may feel obliged to stay at the company (normative commitment). The employees want to retain this favorable group membership. The relationship of CSR and commitment was investigated several times (e.g., Choi and Yu, 2014), and identification mediated this relationship (e.g., Farooq et al., 2014; Islam et al., 2018).

*Hypothesis 5*: CSR is positively related to organizational commitment.

Social identity theory also applies to job satisfaction which is characterized by a pleasurable or positive emotional state resulting from the appraisal of one's job or job experiences (Locke, 1976). The favorable characteristics of a company, e.g., CSR engagement, are associated with prestige and feelings of pride. These feelings evoke job satisfaction (Ellemers et al., 2011). The relationship of CSR and job satisfaction was investigated several times (e.g., Lee et al., 2013; Song et al., 2015), and identification mediated this relationship (e.g., Shin et al., 2016; El Akremi et al., 2018).

*Hypothesis 6*: CSR is positively related to job satisfaction.

The processes underlying social identity theory not only relate to organizational attitudes, but are also associated with behavioral outcomes (Ashforth et al., 2008). When people categorize themselves in terms of their membership of a company engaging in CSR and identify with a socially responsible organization, they are inclined to behave according to the values associated with this group membership (Ellemers et al., 1999). CSR provides a behavioral guideline in terms of citizenship behavior (Lin et al., 2010) or the employees do not want to remain beneficiaries but to contribute on their own by showing OCB (Hansen et al., 2011; Chun et al., 2013). Identification mediated this relationship of CSR and OCB (e.g., Newman et al., 2016; Shen and Benson, 2016; Farooq et al., 2017).

*Hypothesis 7*: CSR is positively related to OCB.

The analysis of the relationships between CSR and employee-related outcomes also allows to determine the magnitude of these relationships.

### Distinguishing Attitudes and Behavior

We distinguish between attitudes and behaviors (Gond et al., 2017), as we hypothesize that CSR affects them differently. As it is easier to influence attitudes than behavior (Ajzen et al., 1980), we hypothesize that CSR is stronger when associated with attitudes (identification, engagement, commitment, job satisfaction, turnover intentions, and attractiveness) than behavioral outcomes (OCB and actual turnover). In their review, Ashforth et al. (2008) point out that identity behaviors are part of the process of identification but are not at the core of their model, where self-definitions and affect followed by beliefs are. Some studies report weaker relationships between CSR and OCB than between CSR and commitment or job satisfaction (Evans et al., 2011; Choi and Yu, 2014; Zhang et al., 2014).

*Hypothesis 8*: The relationship between CSR and employee-related attitudes (identification, engagement, commitment, job satisfaction, turnover intentions, and attractiveness) is significantly stronger than the relationship between CSR and employee-related behavioral outcomes (OCB and actual turnover).

### Distinguishing the Focus of CSR

We hypothesize that the CSR foci [e.g., people, planet and profit according to Elkington (1994)] each have different impacts on employee-related attitudes and citizenship behaviors. The differential relationship of the particular CSR foci and employee-related attitudes and citizenship behaviors is supported by empirical data (Lin et al., 2010; Smith and Langford, 2011; Stites and Michael, 2011). For example, research findings show that the strength of the relationship between CSR and identification depends on the focus of CSR (Farooq et al., 2017). CSR toward the community as well as internal CSR (counted among *people*) showed the highest correlation with identification, whereas CSR toward the environment (counted among *planet*) correlated least. We propose that initiatives with a focus on *people* are more strongly related to all employee-related attitudes and citizenship behaviors under investigation than CSR focusing *planet* and *profit*, as these initiatives directly impact the employees in their workplace (De Roeck and Maon, 2018).

*Hypothesis 9:* Specific foci of CSR (people, planet, profit) are positively associated with employee-related outcomes. The mean effect size varies depending on the CSR focus.

**Figure 1:**
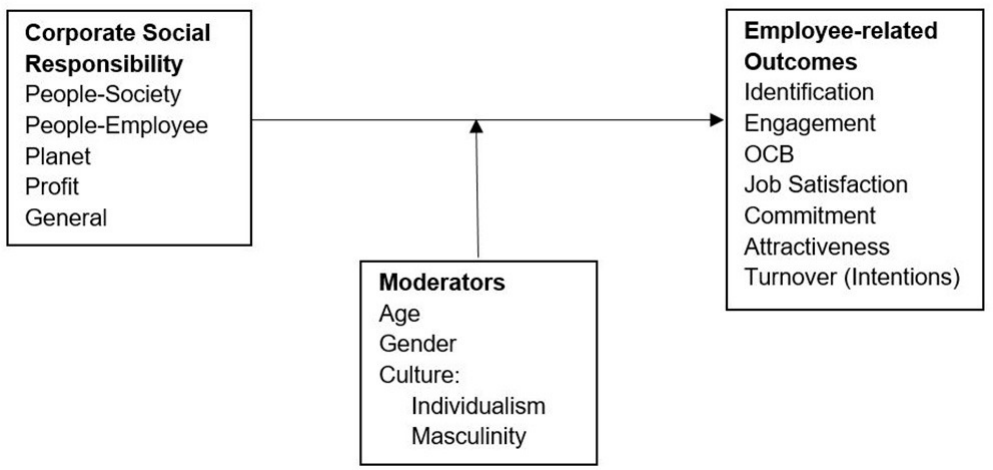
Overview of constructs under investigation.

Figure 1 gives an overview of the constructs under investigation.

### CSR and Social Identity Theory in Organizations

Social identity theory in organizations serves as a theoretical framework to explain the relationship between CSR and identification, which, in turn, is associated with further outcomes such as commitment, job satisfaction and OCB. As we are interested in the magnitude of all relationships of CSR and the outcomes under investigation, we treated identification as an outcome up to now to determine the magnitude of the relationship of CSR and identification. Next, according to social identity theory, we investigate identification as a mediator of the relationship between CSR and the other outcomes. Therefore, we formulate the following hypothesis:

*Hypothesis 10:* CSR and a) employee-related outcomes (engagement, commitment, job satisfaction, attractiveness, and OCB) are mediated by identification.

**Figure 2:**
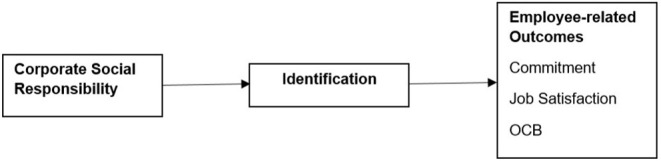
Overview of meta-analytical mediation model.

Figure 2 displays the meta-analytical mediation model.

## Method

### Inclusion Criteria

We defined several inclusion criteria for eligible studies. First, CSR had to be measured on the individual level, for example CSR perceptions. Second, at least one of the following criteria had to be measured: organizational identification, work engagement, attractiveness as a (prospective) employer, turnover (intentions), OCB, organizational commitment or job satisfaction. Third, a correlation between CSR and the employee-related outcome had to be reported. Studies were also included if they provided enough information to compute a correlation or enabled transformation into a correlation, except of regression coefficients (Roth et al., 2018). Fourth, participants had to be employees or prospective employees, more precisely students in their last academic years in their role as job seekers or potential employees. In experimental studies, participants had to be either employees or students. Studies were excluded if the study population were customers. Our sample includes studies from several countries and studies with various research designs.

### Search Strategy

**Figure 3:**
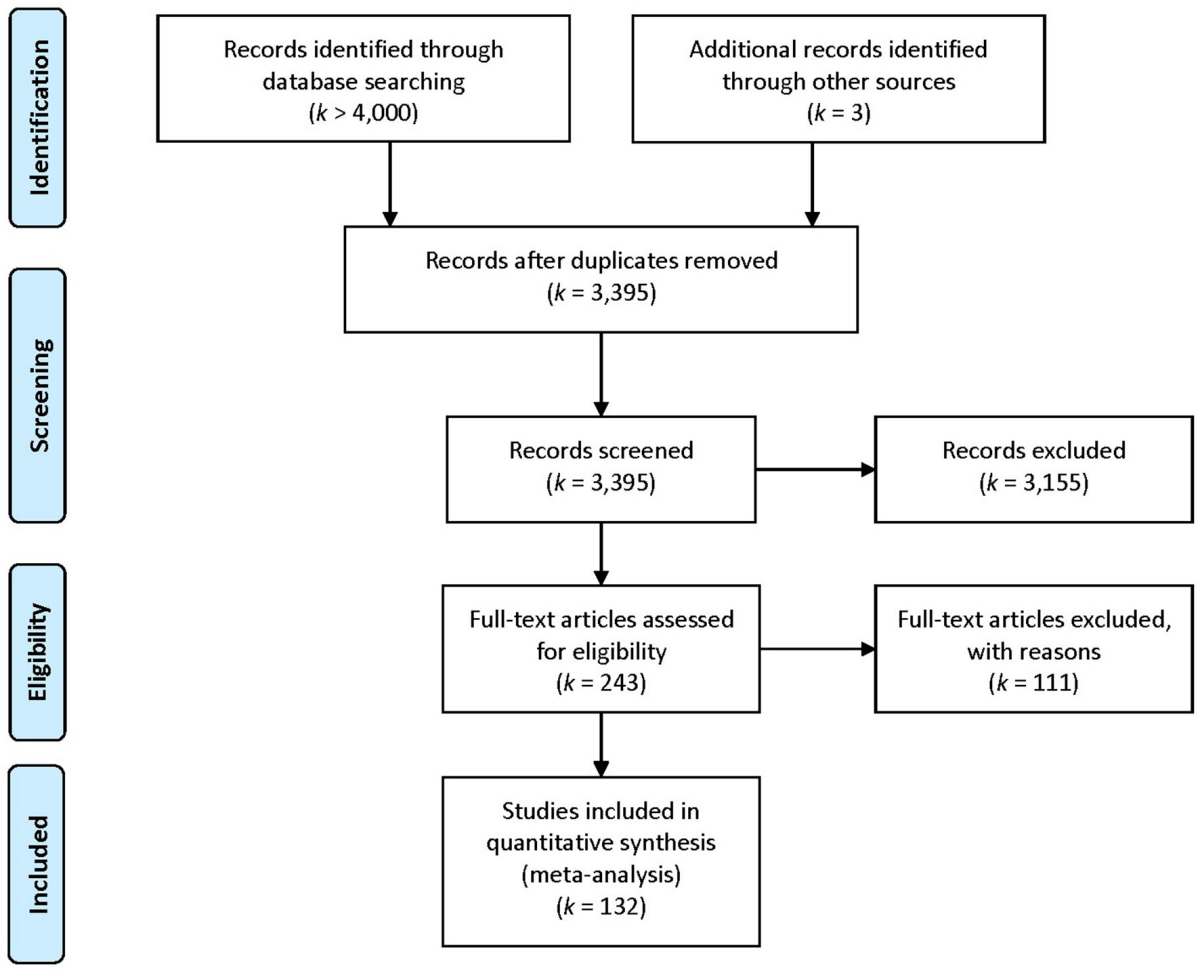
Primary study flow chart depicting numbers of excluded and included articles.

In order to identify potential studies to be included in the meta-analysis, a computer-based search was conducted. The following databases were scanned: PsycINFO, SSCI and EconLit. The key words used were: *corporate social responsibility, social responsibility, socially responsible, corporate responsibility, corporate responsible, CSR, philanthropy, corporate charitable contributions, charitable contributions, corporate citizenship, corporate conscience, corporate donations, environmental performance, social performance, responsible business, greenwashing, corporate sponsorship, identification, engagement, attractiveness, organizational citizenship behavior, OCB, organizational citizenship behavior, contextual performance, prosocial organizational behavior, prosocial behavior, extra-role behavior, commitment, job satisfaction, work satisfaction*, and *employee satisfaction*, whereby we used two brackets to group the key words related to CSR and the employee-related outcomes, AND to link the two brackets and OR within the brackets. The key words have been limited to the title or abstract and, if possible, search results were limited to empirical studies (PsycINFO). Unpublished studies were eligible and contained, for example, dissertation theses (authors were contacted). Some key words were initially included in the search term, but may ultimately not be included in the analysis, because they turned out to be irrelevant during the process (e.g., prosocial behavior did not met the employee/work context criteria). After removing duplicates, 3,398 studies remained for examination. Figure 3 contains a flow chart with details concerning inclusion and exclusion of studies. The most studies were excluded because they did not report CSR or the abbreviation was used otherwise (customer service representative, chemical safety report, etc.). Some publications reported same samples, so the older ones were excluded (*k* = 2). If articles or required data were not available, the authors were contacted. In most cases, unavailable articles were dissertation theses and no author contact information was given in the paper or on faculty homepages. The search was terminated by the end of February 2019. In sum, 132 articles comprising 143 effect sizes were included in the meta-analysis resulting in a total sample size of *N* = 89,396.

### Coding Procedures

#### General Coding Procedures

To validate the coding procedures, eligible studies were coded by two independent coders applying a standardized coding manual. The second coder, a subject matter expert, coded 20% of randomly selected studies and intercoder agreement was assessed. For continuous data, a two-way random single measure intraclass correlation (ICC 2.1) was computed. The ICCs for the variables year of publication, sample size, gender, age, culture, effect size *r* and the reliabilities of the measurement of CSR and the attitudes and citizenship behaviors ranged from 0.92 to 1. Cohen's kappa was computed for categorical data (Cohen, 1968): study design, subject group, and CSR focus and outcome measure and ranged from κ = 0.97 to κ = 1. Disagreements, as reflected by the values of the consistency measures, were very rare and were resolved by discussion.

To assess study quality and to judge its (potential) influences, study design features were coded and investigated as moderators: study design, publication status (published or not) and year of publication.

#### Coding of Moderating Variables

In meta-analysis, all subsample analyses are statistically termed moderator analysis. Following this rationale, distinguishing different outcomes or distinguishing between attitudes and behavior are moderator analyses, although they are not conceptual moderators.

##### Employee-Related Attitudes and Citizenship Behaviors

We classified the employee-related attitudes and behaviors as follows: identification, engagement, attractiveness to (potential) employees, turnover intentions, commitment, job satisfaction (attitudes) and OCB and actual turnover (behavior). Table A (online supplement at https://osf.io/ztdhr/) contains detailed information on the operationalization of all constructs for each study included in the meta-analysis. Attractiveness was measured by organizational attractiveness scales. Turnover was measured by turnover intentions or actual turnover. For the analysis of attitudes and behavior, actual turnover was included in the category *behavior*, whereas turnover intentions were included in the category *attitudes*. In critical cases, we made our decisions based on the content of variables, not the labels: When constructs were named similar to the ones we defined (e.g., stakeholder-company identification), or items were self-developed, we performed an in-depth examination of construct definitions and items in the respective publication. For example, job satisfaction may have been labeled as work satisfaction or employee satisfaction, but all variables were measured using the same pool of questionnaires. Some of the constructs under investigation were multi-dimensional. For example, the construct engagement consists of the subdimensions dedication, vigor and absorption. Some studies reported the one-dimensional higher-order construct, and some reported all subdimensions of a construct (lower-order components). In our analysis, we used the higher-order-constructs.

##### Focus of CSR

As measurement of CSR can focus on different aspects of CSR, the focus of the CSR measurement was registered by using the following categories: people-society, people-employee, planet, profit, general. This category system is based on Elkington's conceptualization named Triple Bottom Line (Elkington, 1994). The category *people*, implied a social orientation of CSR. Because employees are the focus of this meta-analysis, we differentiated between a focus on a company's own employees (people-employee) and a focus on society in general (people-society). *People-society* included the following exemplary terms: ethical, discretionary, legal, philanthropic, CSR to government and CSR to customers. Volunteerism programs were counted among the *people-employee* category. If the focus of a people-focused CSR measure was not clear, *people-society* was coded. *Planet* included environmental aspects of CSR, whereas the category *profit* included economic or financial aspects of CSR. The category *general* was assigned if it focused on multiple aspects of CSR or if no specific focus was identifiable. This categorization does not contradict other authors' CSR conceptualizations (Carroll, 1991; Turker, 2009; El Akremi et al., 2018).

##### Study Design and Population Characteristics

Gender was coded by recording the percentage of males in the study population (or computed from the percentage of women or absolute frequencies). As the majority of research suggests that there are cultural differences of CSR practices and the perception of CSR (Küskü and Zarkada-Fraser, 2004; Dögl and Holtbrügge, 2014; Farooq et al., 2017) and others argue that CSR may be a universal phenomenon (Quazi and O'Brien, 2000), we included culture among the population characteristics variables. Culture was assessed by means of the individualism/collectivism and masculinity/femininity dimensions of culture by Hofstede (2001) which enabled to assign a score between 1 and 100 to each country. These two dimensions are most widely used in the context of CSR and culture (Smith et al., 2011; Hofman and Newman, 2014). High scores indicate an individualistic or masculine culture.

Study design was coded by recording if the study design was a) predictive or concurrent and if b) the study was a survey study, experimental or quasi-experimental study. If the predictor and the criterion were measured simultaneously, the design was concurrent. If there was a time lag between the assessment of the predictor and criterion, the design was predictive. Moreover, the status (published vs. unpublished) and year of publication were recorded.

### Statistical Methods

For this meta-analysis, we applied the meta-analytical methods of Schmidt and Hunter (2015) and chose a random effects model, because systematic effects of study-level influences are assumed and moderating effects will be analyzed. Effect size metrics were correlation coefficients. In order to compute the mean corrected correlation coefficient ρ, effect sizes were weighted by sample size and individually corrected for measurement artifacts, specifically unreliability of the predictor and the criterion. A 95% confidence interval (CI) was computed for the mean correlation ρ and indicated the significance of ρ: the mean effect size is significant if the confidence interval does not include zero.

If data were not reported in the primary studies, we conducted transformations where possible. For example, if *r* was not reported, we transformed Cohen's *d* into *r* using a formula correcting for unequal group sizes (Borenstein et al., 2011). Standardized regression coefficients and standardized coefficients obtained in SEM were not transformed, following the recommendations by Roth et al. (2018). Instead, the authors were contacted and asked if they would provide the required correlations. If constructs were measured by means of single-item-measures, a reliability of α = 0.70 was assigned (Wanous and Hudy, 2001). If correlations were obtained from SEM or confirmatory factor analysis (CFA), we coded the reliability as α = 1, because these correlations already are corrected in terms of measurement error. In unclear cases, such as if a study reported a CFA but used regression to test hypotheses, we concluded that the CFA was only conducted to assess the factor structure and quality of measurement instruments and did not adjust the reliability. In even more unclear cases, we made conservative decisions by assigning α = 1 to not overestimate effects. For artifact correction, the artifact distribution method was used. Several artifact distribution procedures are available. Using the R package psychmeta, we chose an automatic selection of the correction procedure based on the available artifacts and the logical arguments provided to the function (Dahlke and Wiernik, 2019). If studies reported more than one effect size, a composite correlation and reliability was computed as recommended by Schmidt and Hunter to ensure independence of effect sizes (using the Spearman Brown formula for composite reliabilities; 2015), in line with other meta-analyses reporting strategies of averaging, pooling or combining to composites, or reporting a total effect across a set of dependent and/or independent variables (e.g., Riketta, 2002, 2008; Faragher et al., 2013; Klug and Maier, 2015; Feitosa et al., 2020). For some subsample analyses, for example the comparison of attitudes and behavior, several attitudes such as job satisfaction and engagement were combined to ensure independence of effect sizes, and analyzed separately thereafter (subsample analysis outcome).

Heterogeneity was measured by means of the Q-statistic, the credibility interval (CR), variance accounted for by artifacts (% *VE*) and *I*^2^. The Q-statistic assesses heterogeneity among the effect sizes by computing the ratio of total observed variation to the within-study error (Borenstein et al., 2011). A statistically significant Q-value indicates heterogeneity. The 80% credibility interval indicates heterogeneity (with wider intervals indicating heterogeneity; Whitener, 1990). Koslowsky and Sagie (1993) offer a rule of thumb and propose searching for moderating effects, if this interval is broader than *r* = 0.11. Furthermore, the *I*^2^ statistic is reported which indicates the ratio of total variation in study estimates that is due to heterogeneity (Higgins and Thompson, 2002). *I*^2^ ranges from 0 to 100% (Borenstein et al., 2011) and the sample can be regarded as heterogeneous, if this value exceeds 75%.

To investigate moderating effects, two strategies were applied: subsample analysis and meta-regression. If the moderator of interest was a categorical variable, the overall sample was divided into subsamples, which were then analyzed separately. Analyses were computed if subsamples contained at least three datasets. A significant difference was then assessed by computing the value Q_bet_. The total variance Q consists of within-study variance (Q_with_) and between-study variance (Q_bet_). The amount of between-study variance and its statistical significance indicate if the subsamples are statistically different from each other. Further indices and procedures can serve for the interpretation of the moderators (narrowing of the confidence intervals after moderator analysis, increase in %VE and decrease in *I*^2^), but we primarily used the Q_bet_-statistic to assess significance of the moderator variable. If the moderator of interest was a continuous variable, meta-regression (mixed-effects model) was applied which is analogous to multiple regression (Cooper, 2010). Meta-regression involves a regression of the correlations of the primary studies onto the potential moderators, originally proposed by (Glass, 1977).

Mediation was tested using meta-analytical structural equation modeling, more specifically using the two-stage structural equation modeling approach (TSSEM) (Cheung, 2015; Jak, 2015). We pursued a conservative strategy and included only studies that measure CSR, identification, and one of the outcomes. In the first stage, the correlations of the correlation matrix are pooled and then this pooled correlation matrix is used for the structural equation model in stage 2. Only study level variance is estimated for the correlation coefficients, because it was not possible to estimate the full random effects covariance matrix (e.g., Jak, 2015).

Studies must have reported the correlation between CSR and identification and the correlation between identification and at least another outcome (and all intercorrelations) to be included in the TSSEM. The model fit is evaluated using the chi square model of fit, Root Mean Squared Error or Approximation (RMSEA; Steiger and Lind, 1980), and the Comparative Fit Index (CFI).

Publication bias was addressed by means of a trim-and-fill funnel plot (Duval and Tweedie, 2000a,b), a leave-one-out-analysis and Egger's test (Egger et al., 1997). As the probability of publication was higher for manuscripts with significant than non-significant results, meta-analysis is prone to a bias overestimating the mean effect size.

The software R (version 4.1.2) and the packages psychmeta (main analysis, sensitivity analysis and cumulative meta-analysis; Dahlke and Wiernik, 2019), metafor (funnel plot and Egger's test; Viechtbauer, 2010), metaSEM (mediation; Cheung, 2015), and rmeta (forest plot; Lumley, 2012) were used for the computations.

## Results

### Characteristics of the Database

As mentioned above, 132 articles comprising 143 effect sizes were included in the meta-analysis. Table 1 gives an overview of the database's characteristics. With regard to gender and culture, the sample of studies was nearly balanced.

**Table 1:** Study and population characteristics.

**Study characteristics**		**Population characteristics**	
*k*	143	Gender (% male)	53.34
*N*	89,396	[0; 100]	
Sample sizes (range)	47 – 15,184	Mean age	33.94
Publication years	1999 – 2018	[21; 52]	
Publication	Number of studies	Culture^b^	
PublishedUnpublished	137 6	Individualism/collectivism	49.49
Study design		[14; 91]	
Predictive^a^	10	Masculinity/Fem.	51.09
Concurrent	133	[14; 70]	
Study type		Occupation	Number of studies
Survey study	129	Employee	123
Experimental	13	Student^c^	11
Quasi-experiment	1	Country	
Outcomes		Belgium	2
Identification	37	Canada	4
Engagement	11	China	14
OCB	31	France	1
Commitment	68	Germany	5
Job satisfaction	40	Greece	3
Attractiveness	25	India	4
		Israel	2
		Italy	1
		Netherlands	2
		Pakistan	9
		Poland	2
		Portugal	4
		Singapore	3
		South Africa	1
		South Korea	16
		Spain	3
		Taiwan	4
		Thailand	2
		Turkey	6
		UK	4
		USA	17
		Vietnam	1
		Multinational	20

*k, number of effect sizes; N, total number of participants, numbers in square brackets indicate ranges*.

a *mean time lag = 5.69 months*

b *Individualism/Collectivism and Masculinity/Femininity scores of Hofstede (2001) Culture Index (values between 1 and 100). High scores indicate an individualistic/masculine orientation*.

c *students were only included in the analysis of attractiveness*.

### Examination of Differential Influences of CSR on Employee-Related Attitudes and Citizenship Behaviors (Moderators)

In meta-analyses, the examination of variables that explain the heterogeneity of the main effect are statistically termed moderators. Technically, one effect size from each sample is included in and is synthesized to an overall effect, but in this case, an overall effect size would be misleading as this would require to merge, e.g., attitudes and behavior. The subsample analyses of the specific outcomes, attitudes and behaviors as well as the CSR foci were statistically treated as moderator analyses. Not all of these analyses are based on conceptual moderators, rather they are termed moderators following the meta-analytical rationale.

### Differential Influences of CSR on Employee-Related Attitudes and Citizenship Behavior

Following our research question, the primary aim of the study was to investigate how strong the relationships between (perceived) CSR and employee-related attitudes and citizenship behaviors are. The examination of the average effect sizes revealed differences as to the size of the mean corrected effect size. The effect sizes were medium to large ranging from ρ = 0.35 for attractiveness, followed by ρ = 0.41 for OCB and ρ = 0.43 for turnover (intentions), ρ = 0.49 for identification, ρ = 0.52 for job satisfaction and ρ = 0.58 for commitment to ρ = 0.64 for engagement. With rare exceptions (e.g., Ong et al., 2018), data on OCB was obtained from self-report measures. The value of Q_bet_ = 370.64 (*p* < 0.001) indicates that there were differences concerning the outcomes (Table 2). As the confidence intervals did not include zero, all correlations were significantly different from zero. Hypotheses 1–7 are supported.

**Table 2:** Subsample analyses for employee-related outcomes and CSR dimensions.

	***k***	***N***	***r***	***SD_***r***_***	**ρ**	***SD_**ρ**_***	**95% CI**	**80% CR**	**Q**	***I*^2^ (in %)**
**Outcome type (Q**_**bet**_ **=** 169.58^***^)										
Attitude	130	86,125	0.51	0.14	0.58	0.15	0.56; 0.61	0.40; 0.77	633.27^***^	79.63
Behavior	34	15,346	0.31	0.17	0.35	0.18	0.29; 0.42	0.12; 0.59	366.98^***^	91.01
**Outcomes: employee attitudes and citizenship behaviors (Q**_**bet**_ **=** 370.64^***^)										
Identification^a^	37	10,456	0.43	0.13	0.49	0.13	0.44; 0.54	0.33; 0.65	200.24^***^	82.02
Engagement	11	32,554	0.57	0.14	0.64	0.15	0.54; 0.73	0.45; 0.83	104.28^***^	90.41
OCB	31	10,157	0.36	0.18	0.41	0.20	0.34; 0.48	0.16; 0.66	372.62^***^	91.95
Commitment	68	33,965	0.51	0.13	0.58	0.13	0.54; 0.61	0.42; 0.74	254.80^***^	73.71
Job satisfaction	40	29,297	0.46	0.15	0.52	0.15	0.47; 0.57	0.32; 0.71	253.36^***^	84.61
Attractiveness	10	1,582	0.38	0.24	0.43	0.26	0.26; 0.59	0.10; 0.75	104.41^***^	91.38
Turnover	15	10,865	−0.31	0.09	−0.35	0.09	−0.30; −0.40	−0.24; −0.46	67.93^***^	79.39
**CSR focus (Q**_**bet**_ **=** 301.59^***^)										
People-society	50	39,854	0.46	0.11	0.51	0.11	0.48; 0.55	0.38; 0.65	181.67^***^	73.03
People-employee	32	11,393	0.43	0.17	0.49	0.17	0.42; 0.55	0.27; 0.71	269.60^***^	88.50
Planet	15	5,270	0.37	0.15	0.41	0.15	0.33; 0.50	0.22; 0.61	130.70^***^	89.29
Profit	14	3,568	0.43	0.17	0.48	0.17	0.39; 0.58	0.27; 0.70	61.37^***^	78.82
General	103	53,164	0.54	0.17	0.61	0.18	0.57; 0.64	0.38; 0.83	783.35^***^	86.98

*k, number of data sets; N, total sample size; r, mean uncorrected correlation weighted for sample size; SD_r_, standard deviation of r; ρ, mean corrected correlation weighted for sample size and corrected for artifacts due to measurement error; SD_ρ_, standard deviation of ρ; 95% CI, 95% Confidence Interval; 80% CR, 80% Credibility Interval; %VE, percentage of variance accounted for by artifacts; Q, test of homogeneity of effect sizes; I^2^, measure of inconsistency across study findings*.

a *To assess the magnitude of the relation between CSR and identification, we list identification here among the outcomes; later, identification is investigated as a mediator (H4). Turnover, actual turnover and turnover intentions*.

*** *p < 0.001*.

### Relationships Between CSR and Attitudes and Behavior

To investigate if there is a difference between attitudes and behavior, we divided the database into two subsamples—studies measuring attitudes and studies measuring behavioral outcomes. The effect size for the relationship between CSR and attitudes is ρ = 0.58, and for behavior ρ = 0.35 (Table 2). This difference was statistically significant (Q_bet_ = 169.58^***^), so hypothesis 8 was supported, which stated that the relationship between CSR and attitudes is stronger than the relationship between CSR and behavioral outcomes.

### Differential Influence of CSR Focus

**Figure 4:**
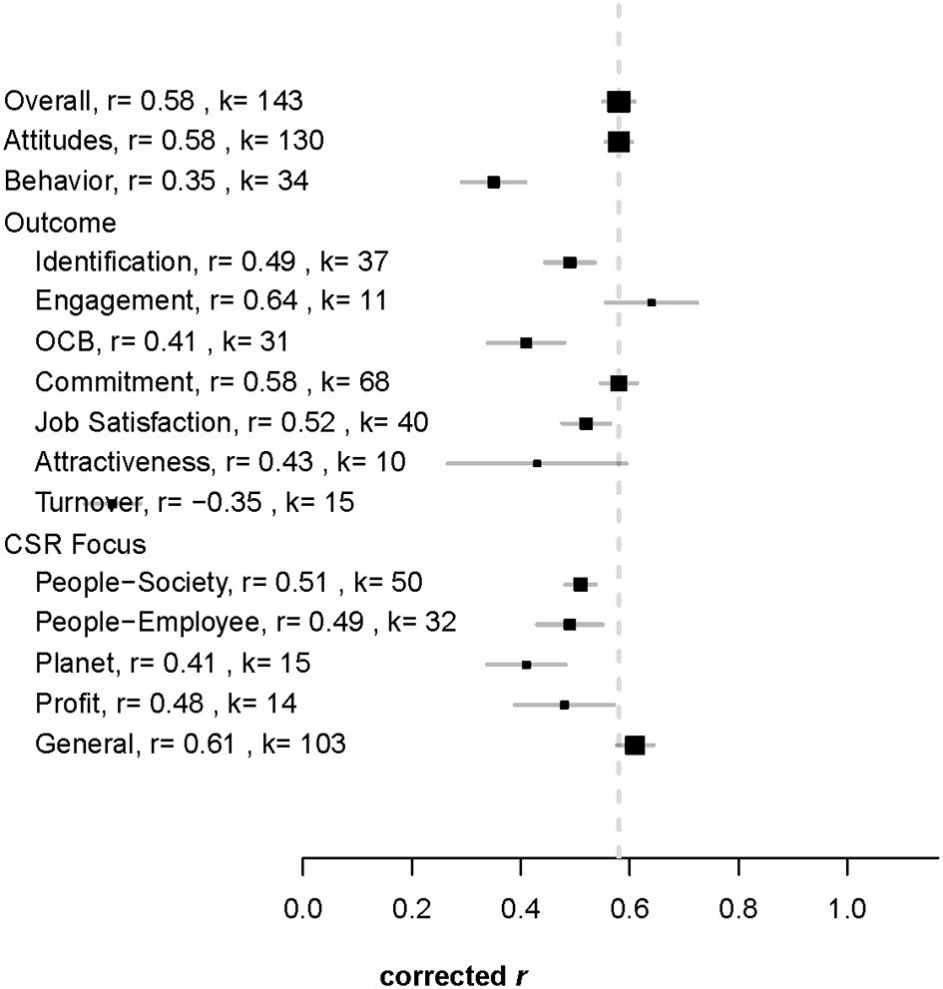
Forest plot displaying effect sizes in relation to the average effect size. Outcomes and CSR dimensions, r, mean corrected effect size; k, number of studies.

Separate analysis of the CSR focus showed that there are differences in the relationship between the particular focus and employee-related attitudes and citizenship behaviors (Q_bet_ = 301.59, *p* < 0.001). For *general*, which combines all different types of CSR, we obtained the largest effect size: ρ = 0.61. Next, we analyzed the specific foci. For *people-society*, we obtained the largest effect sizes of ρ = 0.51. The effect sizes for the other foci ranged from ρ = 0.41 to ρ = 0.49 (Table 2). Hypothesis 9 was supported: The Q-statistic was significant. In Figure 4, effect sizes are displayed visually by means of a forest plot.

### Identification as a Mediator of the Relation Between CSR and Employee-Related Attitudes and Behaviors

Based on a subsample of studies reporting the correlations between CSR and identification (path A), identification and any other outcome (path B) and CSR and the outcome (path C), we specified a meta-analytical structural equation model to test if identification mediated the relationship between CSR and other employee-related attitudes and behavior. The database contained sufficient correlations to investigate commitment (*k* = 7), job satisfaction (*k* = 7) and OCB (*k* = 10) as outcomes in a single model accounting for intercorrelations between all variables under investigation (*k* = 19, *N* = 5,233).

**Figure 5:**
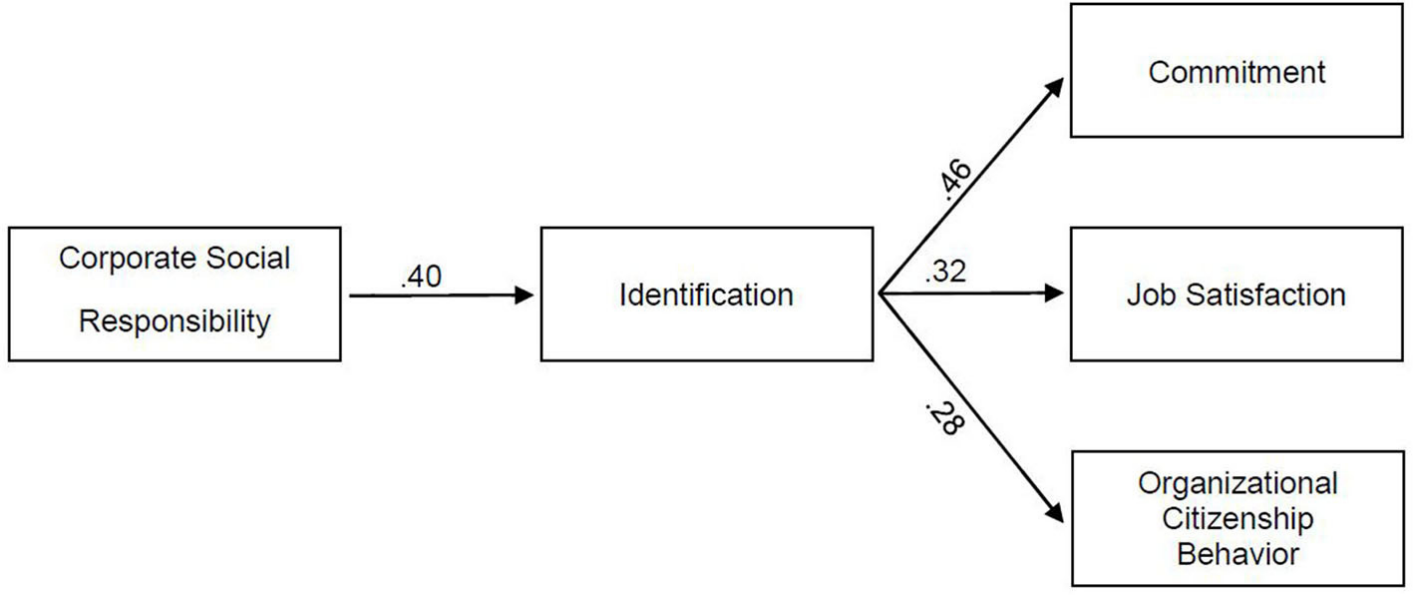
Meta-analytical structural equation model for the relationships between CSR, identification, and commitment, job satisfaction and OCB as employee-related attitudes and behavior.

Figure 5 displays the results of the meta-analytical structural equation model (*X*^2^ = 78.40, *p* = < 0.001, *df* = 6, CFI = 0.92, RMSEA = 0.048). RMSEA and CFI indicate a good model fit, whereas the significant *X*^2^ value does not. In this model, correlations were not corrected for unreliability; the path coefficients are not interpreted the same way as the preceding analyses; the main goal of this analysis is to test mediation by analyzing the indirect effects. In this model, we specified not only the paths from CSR to identification, and from identification to commitment, job satisfaction, and OCB, but also specified direct effect from CSR to commitment, job satisfaction, and OCB, as well as the indirect effects.

All path coefficients were significant. The indirect effect for the relation between CSR and commitment was significant, as it did not contain zero (indirect effect = 0.19, 95% *CI* [0.12; 0.26], direct effect = 0.63, 95% *CI* [0.44; 0.81]). The indirect effects for the relations between CSR and job satisfaction (indirect effect = 0.13, 95% *CI* [0.08; 0.18], direct effect = 0.56, 95% *CI* [0.42; 0.70]) and OCB (indirect effect = 0.11, 95% *CI* [0.05; 0.18], direct effect = 0.27, 95% *CI* [0.15; 0.40]) were statistically significant as well.

Hypothesis 10 is supported for commitment, job satisfaction, and OCB, as identification mediated the relations between CSR and commitment, job satisfaction, and OCB, respectively. There was no sufficient data to investigate the other outcomes.

### Further Analyses

#### Population Characteristics

Moderating effects of the variable gender and age were tested by means of meta-regression. Gender moderated the relationship between CSR and employee-related attitudes and citizenship behaviors, age did not (Table 3). Concerning culture, only masculinity/feminity moderated the relationship between CSR and employee-related attitudes and citizenship behaviors (Table 3), with larger effect sizes in masculine cultures.

**Table 3:** Metaregression (test of continuous moderators).

	**Estimate**	**SE**	***z***	***p***	**95% CI**
Intercept	21.069	16.50	1.277	0.200	−11.27; 54.40
Age	0.004	0.004	1.009	0.313	−0.004; 0.011
Gender	0.003	0.001	2.313	0.021	0.001; 0.006
Publication year	−0.011	0.008	−1.281	0.200	−0.027; 0.006
Culture (Individ.)	−0.000	0.001	−0.322	0.748	−0.003; 0.002
Culture (Masc.)	0.006	0.003	2.176	0.030	0.001; 0.012

*Q (df = 5) = 12.65, p = 0.03, k = 64*.

#### Study Design Characteristics

Study design (predictive vs. concurrent and survey vs. experiment) had a moderating influence on the relationship between CSR and employee-related attitudes and citizenship behaviors (Table 4). Subsamples using concurrent designs resulted in a larger mean effect size than subsamples using predictive designs. Using survey study designs, larger effect sizes were obtained compared to experimental studies (Table 5).

**Table 4:** Subsample analyses for study and population characteristics.

	***k***	***N***	***r***	***SD_***r***_***	**ρ**	***SD_**ρ**_***	**95% CI**	**80% CR**	**Q**	***I*^2^ (in %)**
**Study design (Q**_**bet**_ **=** 18.80^***^)										
Predictive	10	6,348	0.42	0.15	0.48	0.16	0.37; 0.58	0.27; 0.68	72.23^***^	87.54
Concurrent	133	83,048	0.51	0.15	0.59	0.16	0.56; 0.62	0.39; 0.79	697.60^***^	81.37
**Study design (Q**_**bet**_ **=** 23.27^***^)										
Survey study	127	86,711	0.51	0.15	0.59	0.15	0.56; 0.62	0.39; 0.78	645.50^***^	80.48
Experimental^a^	14	2,410	0.29	0.22	0.33	0.23	0.20; 0.45	0.03; 0.62	119.86^***^	89.15
**Status of publication (Q**_**bet**_ **=** 11.53^**^)										
Published	136	88,369	0.51	0.15	0.58	0.16	0.55; 0.61	0.38; 0.78	766.71^***^	82.39
Unpublished	5	752	0.55	0.13	0.63	0.11	0.50; 0.76	0.49; 0.77	10.38^***^	

*k, number of data sets; N, total sample size; r, mean uncorrected correlation weighted for sample size; SD_r_, standard deviation of r; ρ, mean corrected correlation weighted for sample size and corrected for artifacts due to measurement error; SD_ρ_, standard deviation of ρ; 95% CI, 95% Confidence Interval; 80% CR, 80% Credibility Interval; %VE, percentage of variance accounted for by artifacts; Q, test of homogeneity of effect sizes; I^2^, measure of inconsistency across study findings*.

a *contains one quasi-experimental study (N = 412)*.

*** *p < 0.001;*

** *p < 0.01*.

**Table 5:** Subsample analyses for employee-related attitudes and citizenship behaviors and focus of CSR combined.

	***k***	***N***	***r***	***SD_***r***_***	**ρ**	***SD_**ρ**_***	**95% CI**	**80% CR**	**Q**	***I*^2^ (in %)**
**People society (Q**_**bet**_ **=** 64.93^***^)										
Identification	10	3,301	0.33	0.08	0.39	0.07	0.33; 0.45	0.31; 0.47	20.37^**^	55.82
Engagement	6	11,357	0.41	0.07	0.49	0.07	0.42; 0.56	0.40; 0.58	29.76^***^	83.20
OCB	4	823	0.31	0.22	0.37	0.25	0.11; 0.63	0.05; 0.69	33.67^***^	91.09
Commitment	27	22,185	0.48	0.08	0.58	0.08	0.54; 0.61	0.47; 0.68	51.71^***^	49.72
Job satisfaction	16	19,501	0.47	0.15	0.57	0.17	0.48; 0.66	0.35; 0.79	208.90^***^	92.80
Attractiveness	4									
Turnover	4	5,065	−0.33	0.11	−0.38	0.12	−0.26; −0.50	−0.23; −0.53	33.61^***^	91.07
**People-employee (Q**_**bet**_ **=** 115.68^***^)										
Identification	10	3,287	0.37	0.12	0.40	0.11	0.32; 0.48	0.25; 0.55	46.21^***^	80.53
Engagement	2									
OCB	6	1,748	0.37	0.20	0.40	0.21	0.23; 0.57	0.13; 0.67	69.50^***^	92.81
Commitment	19	6,201	0.49	0.17	0.53	0.17	0.45; 0.61	0.31; 0.74	144.03^***^	87.50
Job satisfaction	10	3,582	0.34	0.22	0.36	0.23	0.22; 0.51	0.07; 0.65	150.93^***^	94.04
Attractiveness	2									
Turnover	2									
**General (Q**_**bet**_ **=** 690.07^***^)										
Identification	22	5,715	0.47	0.14	0.50	0.13	0.44; 0.56	0.33; 0.67	134.38^***^	84.37
Engagement	6	21,496	0.65	0.09	0.69	0.07	0.61; 0.76	0.59; 0.78	35.58^***^	85.95
OCB	21	7,479	0.37	0.18	0.39	0.18	0.31; 0.47	0.16; 0.62	275.47^***^	92.74
Commitment	37	11,647	0.52	0.15	0.56	0.15	0.50; 0.61	0.36; 0.75	206.63^***^	82.58
Job Satisfaction	25	9,171	0.44	0.14	0.47	0.14	0.41; 0.53	0.29; 0.64	67.19^***^	64.28
Attractiveness	12	4,619	0.27	0.13	0.27	0.12	0.20; 0.35	0.12; 0.43	72.72^***^	84.87
Turnover	7	2,898	−0.25	0.08	−0.27	0.07	−0.21; −0.33	−0.19; −0.35	14.74^*^	59.29

*k, number of data sets; N, total sample size; r, mean uncorrected correlation weighted for sample size; SD_r_, standard deviation of r, ρ, mean corrected correlation weighted for sample size and corrected for artifacts due to measurement error; SD_ρ_, standard deviation of ρ; 95% CI, 95% Confidence Interval; 80% CR, 80% Credibility Interval; Q, test of homogeneity of effect sizes; I^2^, measure of inconsistency across study findings. Turnover, actual turnover and turnover intentions*.

* *p < 0.05;*

** *p < 0.01;*

*** *p < 0.001*.

Status of publication moderated the relationship between CSR and employee-related attitudes and citizenship behaviors. Unpublished studies reported larger correlations than published studies (Table 4). Results of meta-regression showed that the year of publication did not moderate this relationship (Table 3).

### Sensitivity Analysis

We conducted a sensitivity analysis, more specifically a leave-one-out sensitivity analysis to identify if the findings are driven by a single study, for example studies with large sample sizes. Iteratively, one study at a time is removed to assess the impact of each study on the aggregated effect size. The plots are available in the online supplementary material. One study with a large sample size (*N* = 15,184; Glavas, 2016) had little impact on the aggregated effect size, but it was relatively small (leaving this study out would lower the aggregated effect size by 0.035), so that we decided to keep this study in the database. In summary, the findings are not perfectly robust, yet reasonably robust.

### Assessment of Publication Bias

Schmidt and Hunter (2015) suggest combining the trim and fill funnel plot method and cumulative meta-analysis to assess publication bias, and the application of a third method would substantiate the conclusions concerning publication bias (Schmidt and Hunter, 2015). We followed this rationale by using trim and fill funnel plots, cumulative meta-analysis, and Egger's test.

**Figure 6:**
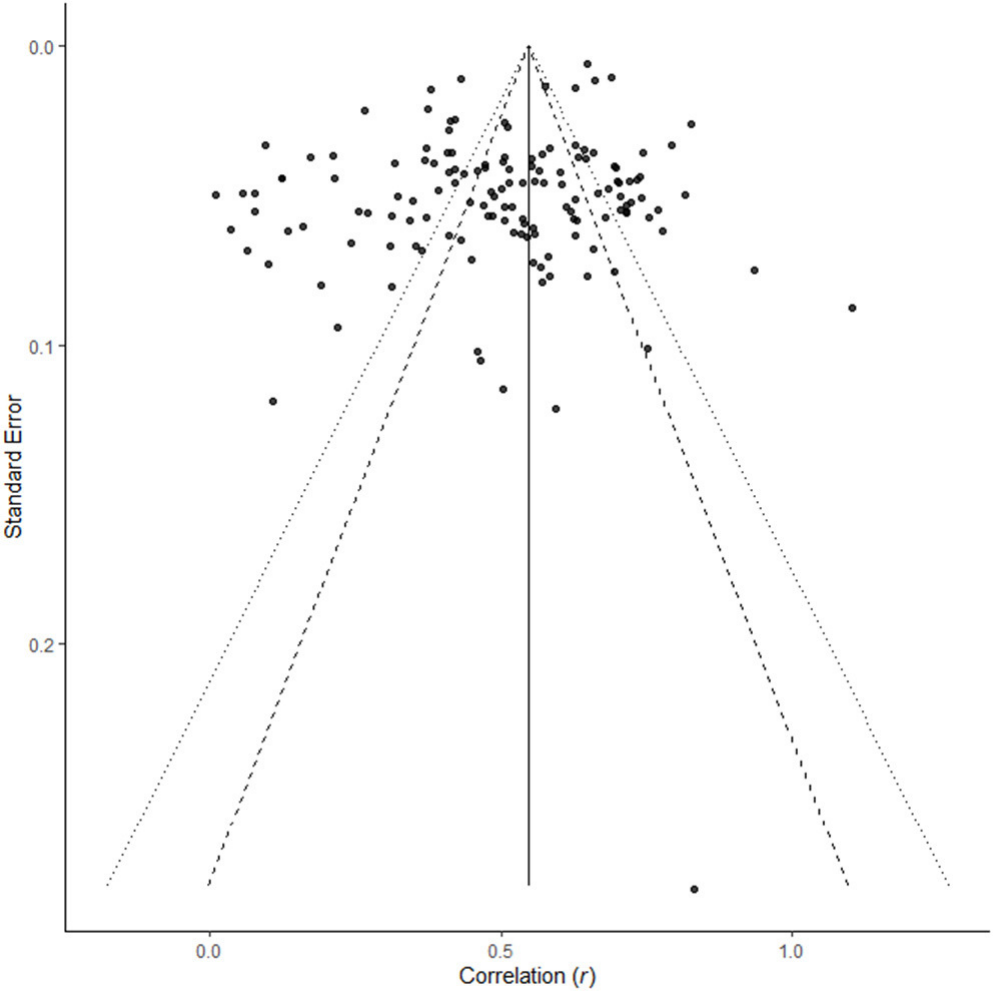
Trim-and-fill funnel plot.

Publication bias was assessed by means of a trim and fill funnel plot (Duval and Tweedie, 2000a,b) which is presented in Figure 6. Visual examination revealed that there is no evidence of the existence of publication bias. Moreover, we conducted a cumulative meta-analysis, which means that studies are added to the analysis one by one, starting with the study with the largest sample size. If the mean effect size becomes smaller when adding the studies with smaller sample sizes, this is an indication for the lack of publication bias (McDaniel, 1990; Borenstein et al., 2011), which was the case in our study. The results of the cumulative meta-analysis are displayed in the online appendix. Egger's test (Egger et al., 1997), a regression-based test which assesses funnel plot asymmetry was not significant (*z* = −1.06, *p* = 0.29), which means the funnel plot is symmetrical, indicating the absence of publication bias.

In summary, the results of the cumulative meta-analysis, the visual examination of the trim-and-fill funnel plot combined with the regression-based test of funnel plot asymmetry lead to the conclusion that publication bias is absent.

## Discussion

CSR is important to employees and positively associated with numerous outcomes such as identification, commitment, job satisfaction, and OCB. In this meta-analysis, we quantified the relationships of CSR with employee-related outcomes, which previously have been proposed in qualitative reviews (Aguinis and Glavas, 2012; Rupp and Mallory, 2015; Glavas, 2016; De Roeck and Maon, 2018). The meta-analytical method allows empirical generalizations concerning CSR (Geyskens et al., 2009). The results of this study which included data of *N* = 89,396 participants show that the effect sizes of the relationships between CSR and employee-related attitudes and citizenship behaviors are medium to large. According to Bosco et al. (2015) benchmarks for the field of applied psychology, effect sizes > ρ = 0.40 are classified as large (medium correlations ranges for attitudes: ρ = 0.18 to 0.39; attitudes and behavior ρ =0.10 to 0.24). The effect sizes for CSR linked with outcomes are large, only the effect size for turnover intentions and turnover is classified as medium. A closer examination of different foci of CSR revealed large correlations, while a combination of different foci and a focus on the society resulted in the largest correlations with employee-related outcomes. Meta-analytical findings in the field of management support the conclusion that effect sizes were large (Paterson et al., 2016): In comparison to major topics in organizational behavior research such as leadership (ρ = 0.35) and training (ρ = 0.25), the effect sizes we found are relatively large. We conclude that CSR is highly relevant to employees and CSR should be assigned an important role in organizations. We investigated employees' perceptions of CSR, so it is important to communicate CSR to employees to ensure they become aware of CSR and not only focus on external communication.

Gender moderated the relations of CSR and the investigated outcomes, whereas age did not. Concerning culture, masculinism/feminism was also identified as a moderator, whereas individualism/collectivism was not. However, the moderating effects were very small and therefore of no practical or managerial relevance. This leads to the conclusion that, based on a large amount of aggregated data, employees rather universally react to CSR in average, regardless of their age, gender, or culture. It is important to note that in specific cases with specific CSR initiatives, there may be gender differences as reported in the literature, for example by Hur et al. (2016) who found gender differences among consumers. Moreover, in a subset of studies, we found that identification mediated the relations of CSR and commitment, job satisfaction, and OCB, respectively.

In this meta-analysis, we investigated the *relationships* of CSR and employee-related outcomes and the nature of most of the included primary studies (*k* = 125) does not allow general causal inferences. However, due to their study designs, some studies provide information about causality for some of the outcomes under investigation. Two longitudinal studies provide evidence on causal effects of CSR: Edwards and Edwards (2013) for identification and intent to quit, and Jones et al. (2014) for attractiveness. A closer examination of the (quasi-) experimental studies (*k* = 14) revealed that most studies (71%) investigated the effect of CSR on attractiveness or turnover intentions (e.g., Zhang and Gowan, 2012; Bode et al., 2015; Joo et al., 2016). The results of three other experimental studies indicate that the effects of CSR on identification, engagement, commitment, job satisfaction, and OCB are causal (Ferreira and Real de Oliveira, 2014; Raub, 2017; Paruzel et al., 2020). In summary, for each outcome at least one study supports the notion of causal effects of CSR.

### Theoretical Implications

Concerning theory, we showed that social identity theory in organizations (Ashforth and Mael, 1989) has the potential to explain the relationships between CSR and a set of employee-related attitudes and citizenship behaviors. Social identity theory is based on the fundamental psychological process of social categorization which explains why CSR changes employees' attitudes (De Roeck and Delobbe, 2012): The membership in the social category of socially responsible companies changes employees' attitudes and behavior. We found a stronger relationship between CSR and attitudinal than behavioral outcomes, and this is consistent with Ashforth et al. (2008) core idea that attitudes are closer to the core identity than behavior. This relation was supported by our data. While attitudes (cognition and emotion) are always involved in the process of identification, behavior is not necessarily involved (Ashforth et al., 2008). Also following major psychological theories, e.g., on work motivation, attitudes precede behavior (Steers et al., 2004; Humphrey et al., 2007). We tested the role of identification as a mediator and found that identification mediated the relationship between CSR and commitment, job satisfaction, and OCB, respectively.

Using a meta-analytical method, we were able to compare the correlations of the three foci (people, planet and profit) and employee-related attitudes and citizenship behaviors to investigate which is most meaningful to employees. We hypothesized that the relationships between the focus of CSR and employee-related attitudes and citizenship behaviors are strongest for CSR with a focus on people. The category people, consists of people-society (external CSR) and people-employees (internal CSR) which indicated the focus of the CSR initiative (initiatives focusing public welfare vs. initiatives specifically addressing employees; Rupp and Mallory, 2015). Effect sizes were significantly larger for the categories people compared to the categories planet, but the effect sizes for profit and people are similar in size. This illustrates that initiatives focusing on the society are highly relevant to employees and probably regarded as most prestigious and distinct compared to initiatives focusing on the natural environment. For the category general, which means that more than one focus was covered by the CSR initiatives, we obtained the largest effect size. This indicates that a combination of several CSR foci and a comprehensive CSR strategy is most effective.

### Implications for Future Research

Given that it is crucial that employees are informed about CSR to be able to perceive CSR, studies about the communication of CSR to employees are needed. For example, here, the content and the medium of CSR communication should be investigated. The content may include more than one CSR focus. Concerning the medium and communication frequency, for example, company newsletters, posters and brochures should be investigated. Moreover, we propose that future studies report information on the degree of participation of employees in CSR, a potential moderator (Bhattacharya et al., 2007; Kim et al., 2010). Degrees of employee participation in CSR range from profound knowledge of CSR programs, to designing them and to taking part in CSR initiatives. Participation could not be analyzed in this meta-analysis due to lack of information in the primary studies. We propose that employee participation in CSR is positively related to the investigated attitudes and citizenship behaviors, which could be explained by the fact that CSR is more salient to them. Participation can be increased by offering all employees the opportunity to submit proposals concerning CSR and to encourage them to take part in CSR initiatives and programs.

The processes underlying social identity theory should be validated in the context of CSR. Concerning social identity theory, in two studies, a mediation by prestige for the relationship between perceived CSR and identification has been investigated (Kim et al., 2010; De Roeck and Delobbe, 2012) but there is a lack of further studies investigating distinctiveness and salience of the out-group. Overall, social identity theory provides a theoretical framework for several employee-related attitudes and behaviors, but also other theoretical explanations should be integrated in comprehensive theory building in future micro-CSR research. The theoretical frameworks of identification, third-party fairness perceptions and meaningfulness do not exclude one another, but rather complement each other.

Apart from this, the results revealed an open research field and we suggest to conduct studies involving multiple perspectives, e.g., using professional CSR rating parallel to measuring CSR perceptions on the individual level in the future. In doing so, we will gain insight if CSR ratings on the company level are in accordance to individual CSR perceptions. This will also answer further research questions, e.g., if CSR initiatives might be perceived as whitewashing by employees, as unmet expectations may result in organizational cynicism (Wilkerson et al., 2008; Evans et al., 2011).

### Practical Recommendations

Regarding the practical value of this meta-analysis, we derive three major implications. First, companies should promote the communication of corporate social issues to employees. To increase identification, commitment, job satisfaction, and OCB, the CSR communication strategy should focus on the central working mechanisms of social identity theory (Ashforth and Mael, 1989), by emphasizing unique features of their own CSR initiatives and by comparing them to those of other companies. The perception of CSR is beneficial to employees, as the results of this meta-analysis show. A mere change of employees' CSR perceptions, e.g., increased awareness or salience of CSR, will be associated positively with employee-related attitudes and citizenship behaviors. Companies can use several communication channels: the intranet, the (employee) newspaper, the notice board, e-mail, staff meetings and social media.

Second, we suggest involving employees in CSR. This will enhance employees' perceived significance of the job (Raub and Blunschi, 2014), the degree to which the job has an impact on other people (Hackman and Oldham, 1975). In this way, CSR gives employees the opportunity to contribute to a higher purpose (Aguinis and Glavas, 2019) and satisfies their need for meaningful existence (Folger et al., 2005; Rupp et al., 2006). Therefore, employees should be given the opportunity to design CSR initiatives or at least submit proposals.

Third, CSR initiatives seem to achieve the best results regarding employees, if they address multiple aspects of CSR. We suggest companies to implement wholesome CSR programs and to focus more than one aspect of CSR by combining people, planet and profit in their CSR strategy. On closer examination of the two societal foci of CSR (see Table 5), the effect sizes of CSR on the outcomes under investigation differ in dependence on the CSR focus, which has either a focus on the employees (people-employee) or on common welfare (people-society). Attractiveness is stronger related to employee-focused CSR, however, job satisfaction is stronger related to people-society. Considering the concept of fairness, we propose the initiatives aiming at society in general and employees to be balanced, so that CSR is not perceived as unfair toward employees (De Roeck et al., 2014; Rupp and Mallory, 2015).

### Limitations

Most subsamples under investigation were heterogeneous, as the ranges of the 80% credibility interval and *I*^2^ show. This means that moderating influences still might be present and have not been detected and that the interpretation of mean effect sizes with wide credibility intervals and *I*^2^ values larger than 75% is limited. When interpreting mean effect sizes, a wide credibility interval (and *I*^2^ larger than 75%) indicate heterogeneity, and the confidence interval provides information about the accuracy of the mean effect size (Whitener, 1990).

Due to some small subsamples, some results should be interpreted with caution. Subsample analyses with imbalanced subsamples (e.g., one subsample consists of a handful of studies, the other one is 10 times as large) can be problematic. The confidence intervals are wider in small subsamples, which make the results not as reliable as large subsamples and the effect sizes are prone to change if more data were included. In our study, this concerns the analysis of study design and population characteristics. The subsamples of subject group and study design were imbalanced (Table 5). Please keep this in mind when interpreting the results containing imbalanced subsamples. However, the hypothesis-relevant subsamples were not imbalanced.

Moreover, studies using self-report measures such as the majority of the studies included in this meta-analysis are often discussed to be subject to common-method bias. Spector and colleagues (Spector et al., 2019) introduced a new approach to this problem and claim that self-report data are not only subject to common method variance which inflates correlations, but are also subject to unshared sources (uncommon method variance) which attenuates correlations. This bias is not caused by self-report data *per se*, it is rather an issue of the measure. As in this meta-analysis the constructs were measured using several different measures in the primary studies, the issue of inflated or attenuated measures might be ruled out. One of the primary studies measured self-reported turnover intentions and actual turnover (Doh et al., 2011). They report similar correlations of CSR and turnover intentions (*r* = −0.28, *N* = 4,250) and actual turnover (*r* = −0.25). Given that other researchers report relatively low correlations of CSR and actual turnover (Bode et al., 2015; Ng et al., 2019), the results seem inconclusive. Yet the study by Doh et al. (2011) was conducted on a large sample and has the advantage that both turnover intentions and actual turnover were measured so that it allows a direct comparison of effect sizes, thus strengthening the conclusion that self-report measures rather not might have inflated the results.

## Conclusion

This meta-analysis includes 132 articles containing 143 studies measuring the relationship between CSR and employee-related outcomes. This quantitative synthesis of research findings on the relationships between CSR and organizational attitudes and citizenship behaviors resulted in mostly large mean effect sizes for the relationships between CSR and identification, engagement, attractiveness, turnover (intentions), commitment, job satisfaction and OCB. All types of CSR (focus on people, planet, or profit) are associated with beneficial employee outcomes, of which initiatives focusing on people (the society) or CSR with more than one focus are associated most strongly with employee-related outcomes. The findings highlight the benefit of employees being informed about CSR. Implications emphasize the need for employee communication of CSR initiatives. Do good and talk about it—with your employees.

## Data Availability Statement

The original contributions presented in the study are included in the supplementary material. The coding sheet, a table containing detailed information on all primary studies included in the meta-analysis, figures depicting the sensitivity analysis, and the assessment of publication bias are available at the OSF repository: https://osf.io/ztdhr/.

## Author Contributions

AP, HK, and GM: conceptualization, writing-review, and editing. AP and HK: methodology (coding and second coding included), formal analysis, and investigation. AP: writing-original draft preparation and resources. HK and GM: supervision. All authors contributed to the article and approved the submitted version.

## Conflict of Interest

The authors declare that the research was conducted in the absence of any commercial or financial relationships that could be construed as a potential conflict of interest.

## References Included in the Meta-Analysis

Abdullah, M., Ashraf, S., and Sarfraz, M. (2017). The organizational identification perspective of CSR on creative performance. The moderating role of creative self-efficacy. *Sustainability* 9, 2125. doi: 10.3390/su9112125
Agarwal, J., Stackhouse, M., and Osiyevskyy, O. (2018). I love that company: Look how ethical, prominent, and efficacious it is—A triadic organizational reputation (TOR) scale. *J. Bus. Ethics* 153, 889–910. doi: 10.1007/s10551-016-3421-2
Akdogan, A. A., Arslan, A., and Demirtaş, Ö. (2016). A strategic influence of corporate social responsibility on meaningful work and organizational identification, via perceptions of ethical leadership. *Procedia - Social and Behavioral Sciences* 235, 259–268. doi: 10.1016/j.sbspro.2016.11.029
Ali, I., Rehman, K. U., Ali, S. I., Yousaf, J., and Zia, M. (2010). Corporate social responsibility influences, employee commitment and organizational performance. *Afr. J. Bus. Manage*. 4, 2796–2801.
Ali, M. A., and Jung, H.-J. (2017). CSR and the workplace attitudes of irregular employees. The case of subcontracted workers in Korea. *Bus. Ethics: Eur. Rev*. 26, 130–146. doi: 10.1111/beer.12146
Alniacik, U., Alniacik, E., and Genc, N. (2011). How corporate social responsibility information influences stakeholders' intentions. *Corp. Soc. Resp. Env. Ma*. 18, 234–245. doi: 10.1002/csr.245
Arendt, S., and Brettel, M. (2010). Understanding the influence of corporate social responsibility on corporate identity, image, and firm performance. *Manage. Dec*. 48, 1469–1492. doi: 10.1108/00251741011090289
Asrar-ul-Haq, M., Kuchinke, K. P., and Iqbal, A. (2017). The relationship between corporate social responsibility, job satisfaction, and organizational commitment. Case of Pakistani higher education. *J. Clean. Prod*.142, 2352–2363. doi: 10.1016/j.jclepro.2016.11.040
Azim, M. T., Diyab, A. A., and Al-Sabaan, S. A. (2014). CSR, employee job attitude and behavior: Saudi Bank experience. *Transylvanian Review of Administrative Sciences* 43, 25–47.
BeBe, K., and Bing, W. (2016). Social responsibility and organisational commitment in local public administration: the moderating role of organisational citizenship behaviour and social bonding. *Mednarodna Revija za Javno Upravo/International Public Administration Review* 14, 13–36. doi: 10.17573/ipar.2016.2-3.01
Besieux, T., Baillien, E., Verbeke, A. L., and Euwema, M. C. (2018). What goes around comes around: The mediation of corporate social responsibility in the relationship between transformational leadership and employee engagement. *Econ. Ind. Democracy* 39, 249–271. doi: 10.1177/0143831X15614914
Bode, C., Singh, J., and Rogan, M. (2015). Corporate social initiatives and employee retention. *Org. Sci*. 26. doi: 10.1287/orsc.2015.1006
Brammer, S., He, H., and Mellahi, K. (2014). Corporate social responsibility, employee organizational identification, and creative effort: The moderating impact of corporate ability. *Group Organ. Manage*. 40, 323–352. doi: 10.1177/1059601114562246
Brammer, S., Millington, A., and Rayton, B. (2007). The contribution of corporate social responsibility to organizational commitment. *Int. J. Hum. Resour. Manage*. 18, 1701–1719.
Bravo, R., Buil, I., Chernatony, L. de, and Martínez, E. (2017). Brand identity management and corporate social responsibility. An analysis from employees' perspective in the banking sector. *J. Bus. Econ. Manage*. 18, 241–257. doi: 10.3846/16111699.2016.1209785
Breitsohl, H., and Ehrig, N. (2017). Commitment through employee volunteering. Accounting for the motives of inter-organisational volunteers. *Appl. Psychol*.66, 260–289. doi: 10.1111/apps.12092
Carmeli, A., Gilat, G., and Waldman, D. A. (2007). The role of perceived organizational performance in organizational identification, adjustment and job performance. *J. Manage. Stud*. 44, 972–992.
Catano, V. M., and Morrow Hines, H. (2016). The influence of corporate social responsibility, psychologically healthy workplaces, and individual values in attracting millennial job applicants. *Can. J. Beh. Sci*. 48, 142–154. doi: 10.1037/cbs0000036
Choi, Y., and Yu, Y. (2014). The influence of perceived corporate sustainability practices on employees and organizational performance. *Sustainability* 6, 348–364. doi: 10.3390/su6010348
Chun, J. S., Shin, Y., Choi, J. N., and Kim, M. S. (2013). How does corporate ethics contribute to firm financial performance? The mediating role of collective organizational commitment and organizational citizenship behavior. *J. Manage*. 39, 853–877. doi: 10.1177/0149206311419662
D'Aprile, G., and Talò, C. (2015). How corporate social responsibility influences organizational commitment: A psychosocial process mediated by organizational sense of community. *Employee Responsibilities and Rights Journal* 27, 241–269. doi: 10.1007/s10672-015-9265-6
Demirel, H., Çalişir, M., and Ergeneli, A. (2013). “Effects of coporate social responsibilty campaigns on employees' organizational commitment in tourism sector,”. The 7th International Days of Statistics and Economics. The 7th International Days of Statistics and Economics, Prague, September 19.
De Roeck, K., and Delobbe, N. (2012). Do environmental CSR initiatives serve organizations' legitimacy in the oil industry? Exploring employees' reactions through organizational identification theory. *J. Bus. Ethics* 110, 397–412.
De Roeck, K., El Akremi, A., and Swaen, V. (2016). Consistency matters! How and when does corporate social responsibility affect employees' organizational identification? *J. Manage. Stud*. 53, 1141–1168. doi: 10.1111/joms.12216
De Roeck, K., and Farooq, O. (2018). Corporate social responsibility and ethical leadership: Investigating their interactive effect on employees' socially responsible behaviors. *J. Bus. Ethics* 151, 923–939. doi: 10.1007/s10551-017-3656-6
De Roeck, K., Marique, G., Stinglhamber, F., and Swaen, V. (2014). Understanding employees' responses to corporate social responsibility: Mediating roles of overall justice and organisational identification. *Int. J. Hum. Resour. Manage*. 25, 91–112.
Dhanesh, G. S. (2014). CSR as organization-employee relationship management strategy: A case study of socially responsible information technology companies in India. *Manage. Commun. Q*. 28, 130–149. doi: 10.1177/0893318913517238
Dhanesh, G. S. (2012). The view from within: Internal publics and CSR. *J. Commun. Manage*. 16, 39–58.
Ditlev-Simonsen, C. D. (2015). The relationship between Norwegian and Swedish employees' perception of corporate social responsibility and affective commitment. *Bus. Soc*. 54, 229–253. doi: 10.1177/0007650312439534
Dögl, C., and Holtbrügge, D. (2014). Corporate environmental responsibility, employer reputation and employee commitment: an empirical study in developed and emerging economies. *Int. J. Hum. Resour. Manage*. 25, 1739–1762. doi: 10.1080/09585192.2013.859164
Doh, J. P., Stumpf, S. A., and Tymon, J. W. G. (2011). Responsible leadership helps retain talent in India. *J. Bus. Ethics* 98, 85–100. doi: 10.1007/s10551-011-1018-3
Du, S., Bhattacharya, C. B., and Sen, S. (2015). Corporate social responsibility, multi-faceted job-products, and employee outcomes. *J. Bus. Ethics* 131, 319–335. doi: 10.1007/s10551-014-2286-5
Edwards, M. R. (2016). The Olympic effect. Employee reactions to their employer's sponsorship of a high-profile global sporting event. *Hum. Resour. Manage*.55, 721–740. doi: 10.1002/hrm.21702
Edwards, M. R., and Edwards, T. (2013). Employee responses to changing aspects of the employer brand following a multinational acquisition: A longitudinal study. *Hum. Resour. Manage*.52, 27–54.
Edwards, M. R., and Kudret, S. (2017). Multi-foci CSR perceptions, procedural justice and in-role employee performance. The mediating role of commitment and pride. *Hum. Resour. Manage. J*. 27, 169–188. doi: 10.1111/1748-8583.12140
El Akremi, A., Gond, J.-P., Swaen, V., Roeck, K. de, and Igalens, J. (2018). How do employees perceive corporate responsibility? Development and validation of a multidimensional Corporate Stakeholder Responsibility Scale. *J. Manage*. 44, 619–657. doi: 10.1177/0149206315569311
Elçi, M., and Alpkan, L. (2009). The impact of perceived organizational ethical climate on work satisfaction. *J. Bus. Ethics* 84, 297–311.
Erdogan, B., Bauer, T. N., and Taylor, S. (2015). Management commitment to the ecological environment and employees: Implications for employee attitudes and citizenship behaviors. *Hum. Relat*. 68, 1669–1691. doi: 10.1177/0018726714565723
Evans, W. R., and Davis, W. D. (2011). An examination of perceived corporate citizenship, job applicant attraction, and CSR work role definition. *Bus. Soc*. 50, 456–480. doi: 10.1177/0007650308323517
Evans, W. R., and Davis, W. (2014). Corporate citizenship and the employee: An organizational identification perspective. *Hum. Perf*. 27, 129–146. doi: 10.1080/08959285.2014.882926
Evans, W. R., Davis, W. D., and Frink, D. D. (2011). An examination of employee reactions to perceived corporate citizenship. *J. Appl. Soc. Psychol*. 41, 938–964.
Evans, W. R., Goodman, J. M., and Davis, W. D. (2011). The impact of perceived corporate citizenship on organizational cynicism, OCB, and employee deviance. *Hum. Perf*. 24, 79–97.
Everding, S. (2016). “Gutes tun, um Personal zu halten - Corporate Social Responsiblity und Mitarbeiterbindung,” [Doing good to hold employees - corporate social responsibility and employee retention]. Master thesis, Bielefeld: Bielefeld University.
Falkowski, P. (2018). “Mein Unternehmen engagiert sich - na und? Zusammenhang zwischen Corporate Social Responsibility und Lebenszufriedenheit sowie prosozialem Verhalten bei Beschäftigten,” [My company is involved in social causes - so what? The relationship between corporate social responsibility and life satisfaction as well as prosocial behavior of employees]. Bachelor thesis, Bielefeld: Bielefeld University.
Farooq, M., Farooq, O., and Jasimuddin, S. M. (2014). ‘Employees response to corporate social responsibility: Exploring the role of employees' collectivist orientation'. *Eur. Manage. J*. 32, 916–927. doi: 10.1016/j.emj.2014.03.002
Farooq, O., Rupp, D. E., and Farooq, M. (2017). The multiple pathways through which internal and external corporate social responsibility influence organizational identification and multifoci outcomes. The moderating role of cultural and social orientations. *Acad. Manage. J*. 60, 954–985. doi: 10.5465/amj.2014.0849
Ferreira, P., and Real de Oliveira, E. (2014). Does corporate social responsibility impact on employee engagement? *Journal of Workplace Learning* 26, 232–247. doi: 10.1108/JWL-09-2013-0070
Gao, Y., and He, W. (2017). Corporate social responsibility and employee organizational citizenship behavior. *Manage. Dec*. 55, 294–309. doi: 10.1108/MD-05-2016-0284
Gao, Y., Zhang, D., and Huo, Y. (2018). Corporate social responsibility and work engagement: testing a moderated mediation model. *J. Bus. Psychol*. 33, 661–673. doi: 10.1007/s10869-017-9517-6
Gaudencio, P., Coelho, A., and Ribeiro, N. (2017). The role of trust in corporate social responsibility and worker relationships. *J. Manage. Development* 36, 478–492. doi: 10.1108/JMD-02-2016-0026
Gkorezis, P., and Petridou, E. (2017). Corporate social responsibility and pro-environmental behaviour. Organisational identification as a mediator. *Eur. J. Int. Manage*. 11, 1–18. doi: 10.1504/EJIM.2017.10001677
Glavas, A. (2010). Effects of corporate citizenship on employees: Why does doing good matter? *Dissertation Abstracts International Section A: Humanities and Social Sciences* 70.
Glavas, A. (2016). Corporate social responsibility and employee engagement: Enabling employees to employ more of their whole selves at work. *Front. Psychol*. 7, 796. doi: 10.3389/fpsyg.2016.00796
Glavas, A., Kelley, K., and Arnold, D. G. (2014). The effects of perceived corporate social responsibility on employee attitudes. *Bus. Ethics Q*. 24, 165–202. doi: 10.5840/beq20143206
Gössling, T. (2011). “CSR and organizational commitment: an empirical study about consequences of CSR on motivation and commitment,” in *Corporate social responsibility and business performance: Theories and evidence about organizational responsibility*, ed. T. Gössling (Cheltenham: Edward Elgar Publishing), 85–102.
Gupta, M. (2017). Corporate social responsibility, employee–company identification, and organizational commitment. Mediation by employee engagement. *Curr. Psychol*. 36, 101–109. doi: 10.1007/s12144-015-9389-8
Hameed, I., Riaz, Z., Arain, G. A., and Farooq, O. (2016). How do internal and external CSR affect employees' organizational identification? A perspective from the group engagement model. *Front. Psychol*. 7. doi: 10.3389/fpsyg.2016.00788
Hamid, M., and Zubair, A. (2016). Corporate social responsibility and organizational commitment among employees in the corporate sector. *Pakistan Business Review* 17, 933–949.
Hansen, S. D., Dunford, B. B., Boss, A. D., Boss, R. W., and Angermeier, I. (2011). Corporate social responsibility and the benefits of employee trust: A cross-disciplinary perspective. *J. Bus. Ethics* 102, 29–45. doi: 10.1007/s10551-011-0903-0
Hofman, P. S., and Newman, A. (2014). The impact of perceived corporate social responsibility on organizational commitment and the moderating role of collectivism and masculinity: Evidence from China. *Int. J. Hum. Resour. Manage*. 25, 631–652.
Hong, G., and Kim, E. (2017). Overcoming country-of-origin image constraints on hiring. The moderating role of CSR. *Asian Bus. Manage*. 16, 253–271. doi: 10.1057/s41291-017-0023-5
Hsieh, S.-C., Chiu, H.-C., Hsieh, Y.-H., Ho, P.-S., Chen, L.-C., and Chang, W.-C. (2016). The perceptions and expectations toward the social responsibility of hospitals and organizational commitment of nursing staff. *J. Nurs. Res*. 24, 249–261. doi: 10.1097/jnr.0000000000000133
Hudson, S., Bryson, D., and Michelotti, M. (2017). Individuals' assessment of corporate social performance, person-organization values and goals fit, job satisfaction and turnover intentions. *Ind. Relat*. 72, 322–344.
Ilkhanizadeh, S., and Karatepe, O. M. (2017). An examination of the consequences of corporate social responsibility in the airline industry. Work engagement, career satisfaction, and voice behavior. *J.Air Transp. Manage*. 59, 8–17. doi: 10.1016/j.jairtraman.2016.11.002
Im, S., Chung, Y., and Yang, J. (2017). Employees' participation in corporate social responsibility and organizational outcomes. The moderating role of Person–CSR Fit. *Sustainability* 9, 28. doi: 10.3390/su9010028
John, A., Qadeer, F., Shahzadi, G., and Jia, F. (2017). Corporate social responsibility and employee's desire. A social influence perspective. *Serv. Ind. J*. 37, 819–832. doi: 10.1080/02642069.2017.1353081
Jones, D. A., Willness, C. R., and Madey, S. (2014). Why are job seekers attracted by corporate social performance? Experimental and field tests of three signal-based mechanisms. *Acad. Manage. J*. 57, 383–404. doi: 10.5465/amj.2011.0848
Joo, Y. R., Moon, H. K., and Choi, B. K. (2016). A moderated mediation model of CSR and organizational attractiveness among job applicants: Roles of perceived overall justice and attributed motives. *Manage. Dec*. 54, 1269–1293. doi: 10.1108/MD-10-2015-0475
Jung, H.-J., and Ali, M. (2017). Corporate social responsibility, organizational justice and positive employee attitudes. In the context of Korean employment relations. *Sustainability* 9, 1992. doi: 10.3390/su9111992
Kim, H. L., Rhou, Y., Uysal, M., and Kwon, N. (2017). An examination of the links between corporate social responsibility (CSR) and its internal consequences. *Int. J. Hospit. Manage*. 61, 26–34. doi: 10.1016/j.ijhm.2016.10.011
Ko, S.-H., Moon, T.-W., and Hur, W.-M. (2018). Bridging service employees' perceptions of CSR and organizational citizenship behavior: The moderated mediation effects of personal traits. *Curr. Psychol*. 37, 816–831. doi: 10.1007/s12144-017-9565-0
Kowal, J., and Roztocki, N. (2015). Do organizational ethics improve IT job satisfaction in the Visegrád Group countries? Insights from Poland. *J. Glob. Inf. Tech. Manage*. 18, 127–145. doi: 10.1080/1097198X.2015.1052687
Kroh, J. (2014). “Corporate social responsibility: How internal and external CSR perceptions influence employee outcomes.” Lissabon: Work Project, NOVA School of Business and Economics.
Krüger, M. C. (2016). “Die Kriteriumsvalidierung einer deutschen Fassung der CStR-Scale,” [Criterion validation of the German version of the CStR-Scale]. Bachelor thesis, Bielefeld: Bielefeld University.
Küskü, F., and Zarkada-Fraser, A. (2004). An empirical investigation of corporate citizenship in Australia and Turkey. *British J. Manage*. 15, 57–72.
Lee, C.-K., Song, H.-J., Lee, H.-M., Lee, S., and Bernhard, B. J. (2013). The impact of CSR on casino employees' organizational trust, job satisfaction, and customer orientation: An empirical examination of responsible gambling strategies. *Int. J. Hospit. Manage* 33, 406–415.
Lee, E. M., Park, S.-Y., and Lee, H. J. (2013). Employee perception of CSR activities: Its antecedents and consequences. *J. Bus. Res*. 66, 1716–1724. doi: 10.1016/j.jbusres.2012.11.008
Lee, K.-P., You, C.-Y., and Bae, J.-Y. (2016). The impact of foreign ownership and control on the organizational identification of host country managers working at MNC subsidiaries. *Int. J. Hum. Resour. Manage*. 28, 1739–1765. doi: 10.1080/09585192.2016.1244105
Lee, S.-H., Lee, T. W., and Lum, C.-F. (2008). The effects of employee services on organizational commitment and intentions to quit. *Personnel Rev*. 37, 222–237.
Lee, S.-Y., and Seo, Y. (2017). Corporate social responsibility motive attribution by service employees in the parcel logistics industry as a moderator between CSR perception and organizational effectiveness. *Sustainability* 9, 355. doi: 10.3390/su9030355
Lee, Y.-K., Kim, Y. “S.” Lee, K. H., and Li, D.-x. (2012). The impact of CSR on relationship quality and relationship outcomes: A perspective of service employees. *Int. J. Hospit. Manage*. 31, 745–756.
Leslie, L. M., Snyder, M., and Glomb, T. M. (2013). Who gives? Multilevel effects of gender and ethnicity on workplace charitable giving. *J. of Appl. Psychol*. 98, 49–62. doi: 10.1037/a0029943
Lin, C.-P., and Liu, M.-L. (2017). Examining the effects of corporate social responsibility and ethical leadership on turnover intention. *Personnel Rev*. 46, 526–550. doi: 10.1108/PR-11-2015-0293
Low, M. P., Ong, S. F., and Tan, P. M. (2017). Would internal corporate social responsibility make a difference in professional service industry employees' turnover intention? A two-stage approach using PLS-SEM. *Glob. Bus. Manage. Res. Int. J*. 9, 24–41.
Maignan, I., Ferrell, O. C., and Hult, G. Tomas (1999). Corporate citizenship: Cultural antecedents and business benefits. *J. Acad. Market. Sci*. 27, 455–469.
McNamara, T. K., Carapinha, R., Pitt-Catsouphes, M., Valcour, M., and Lobel, S. (2017). Corporate social responsibility and employee outcomes. The role of country context. *Bus. Ethics Eur. Rev*. 26, 413–427. doi: 10.1111/beer.12163
Moon, T.-W., Hur, W.-M., Ko, S.-H., Kim, J.-W., and Yoon, S.-W. (2014). Bridging corporate social responsibility and compassion at work Relations to organizational justice and affective organizational commitment. *Career Develop. Int*. 19, 49–72. doi: 10.1108/CDI-05-2013-0060
Mueller, K., Hattrup, K., Spiess, S.-O., and Lin-Hi, N. (2012). The effects of corporate social responsibility on employees' affective commitment: A cross-cultural investigation. *J. Appl. Psychol*. 97, 1186–1200. doi: 10.1037/a0030204
Müller, A. T. (2018). “Experiment zu Wirkmechanismen des Effekts von Corporate Social Responsibility on employee-related outcomes,” [Experiment on working mechanisms of corporate social responsibility on employee-related outcomes]. Master thesis, Bielefeld: Bielefeld University.
Newman, A., Miao, Q., Hofman, P. S., and Zhu, C. J. (2016). The impact of socially responsible human resource management on employees' organizational citizenship behaviour: The mediating role of organizational identification. *Int. J. Hum. Resour. Manage*. 27, 440–455. doi: 10.1080/09585192.2015.1042895
Ng, T. W. H., Yam, K. C., and Aguinis, H. (2019). Employee perceptions of corporate social responsibility: Effects on pride, embeddedness, and turnover. *Personnel Rev*. doi: 10.1111/peps.12294
Ong, M., Mayer, D. M., Tost, L. P., and Wellman, N. (2018). When corporate social responsibility motivates employee citizenship behavior. The sensitizing role of task significance. *Org. Beh. Hum. Dec. Proc*. 144, 44–59.
Ouyang, Y. (2011). Understanding employees' organizational citizenship behaviors through the mediating role of corporate social responsibility. *Int. Res. J. of Appl. Financ*. 2, 29–49.
Ouyang, Y., Cheng, C. H., and Hsieh, C. J. (2010). Does LMX enhance the job involvement of financial service personnel by the mediating roles? *Problems and Perspectives in Management* 8, 174–181.
Panagopoulos, N. G., Rapp, A. A., and Vlachos, P. A. (2016). I think they think we are good citizens. Meta-perceptions as antecedents of employees' reactions to corporate social responsibility. *J. Bus. Res*. 69, 2781–2790. doi: 10.1016/j.jbusres.2015.11.014
Paruzel, A., Danel, M., and Maier, G. W. (2020). Scrutinizing social identity theory in corporate social responsibility: An experimental investigation. *Front. Psychol. 11*. doi: 10.3389/fpsyg.2020.580620
Peloza, J., Hudson, S., and Hassay, D. N. (2009). The marketing of employee volunteerism. *J. Bus. Ethics* 85, 371–386. doi: 10.1007/s10551-008-9734-z
Pérez, F. J., Romeo, M., and Yepes-Baldó, M. (2018). The corporate social responsibility policies for the inclusion of people with disabilities as predictors of employees' identification, commitment and absenteeism. *Anales de Psicologia* 34, 101. doi: 10.6018/analesps.34.1.237231
Peterson, D. K. (2004). The relationship between perceptions of corporate citizenship and organizational commitment. *Bus. Soc*. 43, 296–319. doi: 10.1177/0007650304268065
Raub, S. (2017). When employees walk the company talk. The importance of employee involvement in corporate philanthropy. *Hum. Resour. Manage*. 56, 837–850. doi: 10.1002/hrm.21806
Raub, S., and Blunschi, S. (2014). The power of meaningful work: How awareness of CSR initiatives fosters task significance and positive work outcomes in service employees. *Corn. Hosp. Q*. 55, 10–18. doi: 10.1177/1938965513498300
Rayton, B. A., Brammer, S. J., and Millington, A. I. (2015). Corporate social performance and the psychological contract. *Group Org. Manage*. 40, 353–377. doi: 10.1177/1059601114561476
Rego, A., Leal, S., Cunha, M. P., Faria, J., and Pinho, C. (2010). How the perceptions of five dimensions of corporate citizenship and their inter-inconsistencies predict affective commitment. *J. Bus. Ethics* 94, 107–127.
Rupp, D. E., Shao, R., Skarlicki, D. P., Paddock, E. L., Kim, T.-Y., and Nadisic, T. (2018). Corporate social responsibility and employee engagement: The moderating role of CSR-specific relative autonomy and individualism. *J. Org. Beh*. 39, 559–579. doi: 10.1002/job.2282
Rupp, D. E., Shao, R., Thornton, M. A., and Skarlicki, D. P. (2013). Applicants' and employees' reactions to corporate social responsibility: The moderating effects of first-party justice perceptions and moral identity. *Personnel Psychol*. 66, 895–933. doi: 10.1111/peps.12030
Senasu, K., and Virakul, B. (2015). The effects of perceived CSR and implemented CSR on job-related outcomes: An HR perspective. *Journal of East-West Business* 21, 41–66.
Shao, B., Cardona, P., Ng, I., and Trau, R. N. C. (2017). Are prosocially motivated employees more committed to their organization? The roles of supervisors' prosocial motivation and perceived corporate social responsibility. *Asia Pacific J. Manage*. 34, 951–974. doi: 10.1007/s10490-017-9512-5
Shaoling, L. (2013). Corporate social responsibilities (CSR) in China: Evidence from manufacturing in Guangdong province. *Dissertation Abstracts International Section A: Humanities and Social Sciences* 73.
Shen, J., and Benson, J. (2016). When CSR is a social norm: How socially responsible human resource management affects employee work behavior. *J. Manage*. doi: 10.1177/0149206314522300
Shen, J., and Jiuhua Zhu, C. (2011). Effects of socially responsible human resource management on employee organizational commitment. *Int. J. Hum. Resour. Manage*. 22, 3020–3035.
Shin, I., Hur, W.-M., and Kang, S. (2016). Employees' perceptions of corporate social responsibility and job performance: A sequential mediation model. *Sustainability* 8. doi: 10.3390/su8050493
Shin, I., Hur, W.-M., Kim, M., and Kang, S. (2017). Hidden roles of CSR. Perceived corporate social responsibility as a preventive against counterproductive work behaviors. *Sustainability* 9, 955. doi: 10.3390/su9060955
Song, H. J., Lee, H.-M., Lee, C.-K., and Song, S.-J. (2015). The role of CSR and responsible gambling in casino employees' organizational commitment, job satisfaction, and customer orientation. *Asia Pacific J. Tour. Res*. 20, 455–471. doi: 10.1080/10941665.2013.877049
Soobramoney, J., and Ledimo, O. (2016). Exploring corporate social responsibility and organisational commitment within a retail organisation. *Risk Governance and Control: Financial Markets and Institutions* 6, 132–140.
Stites, J. P., and Michael, J. H. (2011). Organizational commitment in manufacturing employees: Relationships with corporate social performance. *Bus. Soc*. 50, 50–70. doi: 10.1177/0007650310394311
Story, J., and Neves, P. (2015). When corporate social responsibility (CSR) increases performance: Exploring the role of intrinsic and extrinsic CSR attribution. *Bus. Ethics Eur. Rev*. 24, 111–124. doi: 10.1111/beer.12084
ter Hoeven, Claartje L., and Verhoeven, Joost W. M. (2013). 'Sharing is caring': Corporate social responsibility awareness explaining the relationship of information flow with affective commitment. *Corp. Comm*. 18, 264–279.
Thang, N. N., and Fassin, Y. (2017). The impact of internal corporate social responsibility on organizational commitment. Evidence from Vietnamese service firms. *Journal of Asia-Pacific Business* 18, 100–116.
Turker, D. (2009). How corporate social responsibility influences organizational commitment. *J. Bus. Ethics* 89, 189–204.
Tziner, A., Oren, L., Bar, Y., and Kadosh, G. (2011). Corporate social responsibility, organizational justice and job satisfaction: How do they interrelate, if at all? *Revista de Psicolog*í*a del Trabajo y de las Organizaciones* 27, 67–72. doi: 10.5093/tr2011v27n1a7
Valentine, S., and Godkin, L. (2017). Banking employees' perceptions of corporate social responsibility, value-fit commitment, and turnover intentions. Ethics as social glue and attachment. *Employee Responsibilities and Rights Journal* 29, 51–71. doi: 10.1007/s10672-017-9290-8
Vitell, S. J., Ramos, E., and Nishihara, C. M. (2010). The role of ethics and social responsibility in organizational success: A Spanish perspective. *J. Bus. Ethics* 91, 467–483.
Vlachos, P. A., Panagopoulos, N. G., and Rapp, A. A. (2014). Employee judgments of and behaviors toward corporate social responsibility: A multi-study investigation of direct, cascading, and moderating effects. *J. Org. Beh*. 35, 990–1017. doi: 10.1002/job.1946
Vlachos, P. A., Panagopoulos, N. G., Bachrach, D. G., and Morgeson, F. P. (2017). The effects of managerial and employee attributions for corporate social responsibility initiatives. *J. Org. Beh*. 38, 1111–1129. doi: 10.1002/job.2189
Vlachos, P. A., Panagopoulos, N. G., and Rapp, A. A. (2013). Feeling good by doing good: Employee CSR-induced attributions, job satisfaction, and the role of charismatic leadership. *J. Bus. Ethics* 118, 577–588. doi: 10.1007/s10551-012-1590-1
Wang, W., Fu, Y., Qiu, H., Moore, J. H., and Wang, Z. (2017). Corporate social responsibility and employee outcomes. A moderated mediation model of organizational identification and moral identity. *Front. Psychol*. 8, 855. doi: 10.3389/fpsyg.2017.01906
Wong, A., and Hong Gao, J. (2014). Exploring the direct and indirect effects of CSR on organizational commitment. *Int. J. Cont. Hosp. M*. 26, 500–525. doi: 10.1108/IJCHM-05-2013-0225
Yoon, D., Jang, J., and Lee, J. (2016). Environmental management strategy and organizational citizenship behaviors in the hotel industry. *Int. J. Cont. Hosp. M*. 28, 1577–1597. doi: 10.1108/IJCHM-10-2014-0498
Zhang, L., and Gowan, M. A. (2012). Corporate social responsibility, applicants' individual traits, and organizational attraction: A person–organization fit perspective. *J. Bus. Psychol*. 27, 345–362.
Zhang, M., Di Fan, D., and Zhu, C. J. (2014). High-performance work systems, corporate social performance and employee outcomes: Exploring the missing links. *J. Bus. Ethics* 120, 423–435. doi: 10.1007/s10551-013-1672-8
Zhang, A., Feng, R., and Xue, Q. (2014). “Relationships among work satisfaction, organizational citizenship behaviors, and work achievements, based on corporate social responsibility,” in *International Symposium on Humanistic Management and Development of New Cities and Towns (ISHMD 2014)*, ed. H. Chen, J. Zhang, and L. Hale (Marietta, USA: Amer Scholars Press), 85–96.
Zientara, P., Kujawski, L., and Bohdanowicz-Godfrey, P. (2015). Corporate social responsibility and employee attitudes: evidence from a study of Polish hotel employees. *J.Sust. Tour*. 23, 859–880. doi: 10.1080/09669582.2015.1019511
